# Artificial Intelligence in Fracture Diagnosis on Radiographs: Evidence, Pitfalls, and Pathways for Clinical Integration (2020–2025)

**DOI:** 10.7759/cureus.93124

**Published:** 2025-09-24

**Authors:** Mohammed K Elbahi, Abubakr Muhammed, Mohammed Fadlelmola Abdalla Mohamednour, Fatima S Mukhtar

**Affiliations:** 1 Department of Surgery, Faculty of Medicine, Al Neelain University, Khartoum, SDN; 2 Department of Surgery, University of Gezira, Madani, SDN; 3 Department of Internal Medicine, Al Neelain University, Khartoum, SDN; 4 Department of Neurological Surgery, Al Neelain University, Khartoum, SDN

**Keywords:** artificial intelligence, deep learning, emergency imaging, fracture, radiograph

## Abstract

Missed fractures remain one of the most frequent sources of diagnostic errors in emergency departments, often leading to delayed treatment, morbidity, and increased healthcare costs. Artificial intelligence (AI), particularly deep learning systems, has been increasingly investigated as an adjunct for musculoskeletal imaging. Over the past five years, multiple studies have evaluated the diagnostic performance, clinical utility, and limitations of AI in fracture detection.

This article is a narrative synthesis of literature published from 2020 to 2025, focusing on systematic reviews, meta-analyses, and high-quality prospective studies addressing AI-assisted fracture diagnosis on radiographs and other imaging modalities. Key themes examined include diagnostic accuracy, anatomical and modality-specific performance, real-world deployment, regulatory approvals, and remaining challenges to integration.

The results of the current review showed that decent meta-analyses demonstrated pooled sensitivity and specificity above 90%, showing AI performance comparable to that of radiologists. The strongest results were observed in extremity fractures (wrist, ankle, shoulder), while performance was more variable in ribs and spine. Reader studies confirmed that AI assistance improved radiologists’ sensitivity by 6-8% without loss of specificity. Real-world deployments in emergency departments showed modest reductions in reporting discrepancies and patient length of stay. Commercial platforms, such as OsteoDetect and BoneView, received U.S. Food and Drug Administration (FDA) and Conformité Européenne(CE) approvals, and draft guidance from the National Institute for Health and Care Excellence (NICE) in 2024 recommended AI fracture detection in urgent care. However, challenges persist, including dataset bias, limited generalizability, interpretability, and uncertain patient-centered outcomes.

Several studies have reported that AI may serve as a reliable adjunct in fracture diagnosis, with diagnostic accuracy approaching that of radiologists and potential improvements in clinical workflows when used as an assistive tool. Nevertheless, safe integration is best supported by regulatory approval (FDA, CE, NICE) and, where appropriate, supplemented by external validation, robust datasets, and transparent reporting to ensure performance across different clinical environments. Future directions include multi-modal AI, adaptive learning, pediatric-focused applications, and strengthened real-world evidence from retrospective outcome evaluations and post-market surveillance.

## Introduction and background

A fracture diagnosis remains one of the most frequent and critical tasks in both emergency and orthopedic imaging. Despite technological progress in radiology, missed fractures continue to represent a major source of diagnostic error, accounting for approximately 1-5% of emergency radiograph interpretations in the United States--nearly 20,000-100,000 missed cases annually [1,2]. These errors result in increased morbidity, prolonged hospital stays, malpractice claims, and elevated healthcare costs [3]. Plain radiographs remain the frontline modality, but interpretation challenges--subtle fracture lines, overlapping anatomy, and heavy workloads--frequently lead to diagnostic discrepancies, particularly in high-volume or overnight settings [4].

Over the past five years, artificial intelligence (AI), especially deep learning-based convolutional neural networks (CNNs), has emerged as a valuable adjunct in musculoskeletal imaging. Meta-analyses published between 2022 and 2024 consistently report pooled sensitivities and specificities above 90% [5-7], confirming AI’s potential as a support tool. Reader studies show improved sensitivity without loss of specificity when radiologists use AI [8], while real-world evaluations demonstrate fewer reporting discrepancies and reduced emergency department length of stay [9]. Several platforms, such as BoneView (Gleamer, Paris, France), have achieved Conformité Européenne (CE) marking and U.S. Food and Drug Administration (FDA) clearance [10,11], and the National Institute for Health and Care Excellence (NICE) issued draft guidance in 2024 recommending AI for fracture detection [12].

Yet, challenges remain: dataset bias, limited generalizability, and workflow integration barriers. External validation reveals significant variability across institutions [13]. New datasets, such as GRAZPEDWRI-DX (2022), have improved pediatric applications, but broader validation is still required [14].

This review is a narrative synthesis of evidence from recent meta-analyses and systematic reviews published between 2020 and 2025, summarizing the current state of AI in fracture diagnosis and its clinical integration pathways.

## Review

Methods

Search Strategy and Study Selection

This review was conducted as a narrative synthesis of published evidence on artificial intelligence for fracture detection in radiographs. We performed a structured search of PubMed/MEDLINE, Embase, Web of Science, Scopus, and the Cochrane Library for English-language articles published between January 1, 2020, and August 31, 2025.

Search strategy: Controlled vocabulary (MeSH and EMTREE) and free-text terms combining concepts for fracture, radiograph, X-ray, artificial intelligence, deep learning, convolutional neural network, computer-aided detection, and triage were used. A sample PubMed search string is provided in Supplementary Material 1.

Eligibility Criteria

We included (a) systematic reviews and meta-analyses reporting pooled diagnostic performance of AI for fracture detection on radiographs, (b) prospective deployments or impact studies in urgent and emergency care, (c) major benchmarking datasets relevant to radiographic fracture detection, and (d) regulatory or health technology assessment documents related to AI fracture detection. We excluded CT-only studies, segmentation-only tasks, animal or phantom studies, non-English articles, and conference abstracts without full text.

Selection Process

Two reviewers independently screened titles/abstracts and full texts. Disagreements were resolved by consensus. For each included study, we extracted data on study design, anatomy, dataset size, validation type (internal vs. external), pooled sensitivity, specificity, and area under the curve (AUC), as well as funding/conflict of interest disclosures.

Risk of Bias

We qualitatively assessed risk of bias, noting whether included meta-analyses applied QUADAS-2 (Quality Assessment of Diagnostic Accuracy Studies-2) or PROBAST (Prediction model Risk of Bias Assessment Tool). We acknowledge that restricting the study to English-language publications and the purposive inclusion of clinically relevant studies may introduce a bias.

Diagnostic accuracy: evidence from meta-analyses

Over the past several years, several systematic reviews and meta-analyses have evaluated the diagnostic performance of AI systems in fracture detection. These studies provide strong evidence that AI achieves accuracy comparable to, and sometimes exceeding, that of human readers.

Kuo et al. reviewed 42 studies involving AI for fracture detection across radiographs and CT, reporting pooled sensitivities of 92% (internal) and 91% (external), with a specificity of 91% [5]. Jung et al. reported pooled sensitivity and specificity of 92% and 91%, respectively, but noted reduced performance in spine imaging [6]. Nowroozi et al. found >90% sensitivity and specificity in musculoskeletal radiographs, while highlighting substantial heterogeneity and risk of bias [7].

**Table 1:** Summary of meta-analyses

Meta-analysis	Year	Studies	Sensitivity	Specificity	Notes
Kuo et al. (Radiology) [5]	2022	42	92% (internal), 91% (external)	91%	Comparable to radiologists; radiographs + CT
Jung et al. (PLOS Digital Health) [6]	2024	66	92%	91%	Better for extremities than spine
Nowroozi et al. (Clinical Radiology) [7]	2024	100	>90%	>90%	High accuracy, but heterogeneity present

Together, these meta-analyses suggest that AI has matured into a potentially reliable diagnostic adjunct in fracture detection. While its performance generally parallels that of radiologists, several reader studies and systematic reviews (e.g., Guermazi et al. and Oppenheimer et al.) indicate that its greatest value is realized when AI is integrated as a supportive tool--enhancing reader sensitivity, reducing oversight errors, and promoting more standardized interpretation across clinical environments [8,15].

Performance by anatomy and modality

AI performance varies considerably across different anatomical regions and imaging modalities. The strongest results have been observed in extremity fractures, including the wrist, hand, shoulder, and ankle, where AI models trained on large datasets, such as MURA, consistently achieve sensitivity and specificity above 90% [6,7,16]. Convolutional neural networks (CNNs) remain the backbone of most current fracture detection systems and have demonstrated robust performance across musculoskeletal imaging tasks. More recently, transformer-based models have been applied and have shown promise, particularly in identifying proximal femur fractures in elderly patients [7]. Pediatric fracture diagnosis represents a unique challenge due to growth plates and evolving anatomy; however, the release of the GRAZPEDWRI-DX dataset has enabled significant advances in wrist fracture detection in children [14]. In contrast, vertebral fractures remain more difficult to detect, as overlapping anatomical structures reduce AI sensitivity, underscoring the need for region-specific optimization [6].

Reader-aid and real-world impact

AI models have demonstrated strong standalone diagnostic performance, but their true clinical value lies in supporting human readers and improving workflow efficiency. Controlled reader studies consistently show that AI assistance improves radiologists’ sensitivity in fracture detection without compromising specificity. For example, Guermazi et al. demonstrated that when residents and junior radiologists used AI support, their fracture detection sensitivity increased by 10.4%, average reading time was reduced, and specificity remained stable [8]. Similarly, Oppenheimer et al. reported that AI tools helped reduce oversight errors and improved diagnostic confidence in musculoskeletal trauma cases [15].

Beyond experimental studies, early prospective rollouts suggest that AI contributes to modest improvements in patient care pathways. Herpe et al. found that AI deployment reduced reporting discrepancies between initial and final radiology reports and shortened emergency department length of stay by about 5% [9]. Institutional endorsement is also increasing: the UK’s NICE issued draft guidance in 2024 recommending platforms such as BoneView and RBfracture (Radiobotics, Copenhagen, Denmark) for urgent care [12].

Generalizability and external validation

Generalizability remains one of the major challenges in implementing AI for fracture detection. Models often demonstrate high accuracy in internal validation but suffer performance drops in new hospitals or imaging systems due to dataset shift [13]. Factors include differences in imaging protocols, population demographics, and disease prevalence. Variation in prevalence directly influences model calibration and predictive values: low-prevalence settings can reduce positive predictive value despite strong sensitivity and specificity, while high-prevalence cohorts may artificially inflate apparent accuracy or promote overfitting. External validation studies frequently confirm these performance declines, highlighting the importance of rigorous multicenter testing.

Meta-analyses emphasize risk of bias and heterogeneity in published studies [7]. Small sample sizes, limited anatomical coverage, and non-representative training datasets undermine reproducibility. To address these limitations, researchers increasingly employ multicenter datasets, domain adaptation techniques, and prospective validation in clinical workflows. Without these safeguards, AI models risk overfitting and failing to generalize in routine practice [17].

Datasets and benchmarks

Large, well-annotated imaging datasets are essential to developing and benchmarking AI for fracture detection. Benchmark datasets provide a shared reference point for reproducibility and performance comparison across studies. The Stanford MURA dataset (2018) includes over 40,000 upper-extremity radiographs collected from a single U.S. academic hospital [17]. It has been widely used to train and validate deep learning algorithms and remains one of the most cited resources in this field, though it is limited by a single-institution origin and restricted demographic diversity. For pediatrics, the GRAZPEDWRI-DX dataset contributed more than 20,000 wrist radiographs from an Austrian center [14]. This dataset has enabled significant progress in pediatric fracture detection, an area that was previously underrepresented, but it too reflects the demographic and institutional constraints of its source population. Together, these benchmarks have accelerated research and reproducibility, yet they also underscore the importance of prospective curation, standardized labeling practices, and systematic equity testing to strengthen the fairness and external validity of AI systems across diverse clinical environments.

Regulation and commercial tools

The clinical adoption of AI for fracture detection has accelerated in recent years. OsteoDetect was the first FDA-approved AI system for fracture detection, receiving De Novo clearance in 2018 for wrist fractures [11]. BoneView followed with CE marking in Europe and FDA 510(k) clearance in 2022-2023, later extended to pediatric use [10,11]. NICE draft guidance in 2024 endorsed BoneView and RBfracture for urgent and emergency care, marking a significant institutional milestone [12]. These regulatory approvals are critical for establishing safety, enabling reimbursement, and fostering clinician trust. Both FDA and NICE guidance emphasize the importance of post-market surveillance to ensure that AI systems continue to perform safely and effectively across different patient populations and clinical settings.

Pitfalls and limitations

Despite progress, AI faces major limitations. Most systems to date have been trained on single-institution datasets, such as the Stanford MURA dataset [17] and the GRAZPEDWRI-DX pediatric dataset [14], which introduces sampling bias and limits generalizability. In contrast, some industry-led tools (e.g., BoneView) have undergone multi-institutional validation across several European and U.S. centers, illustrating how academic and commercial groups approach the problem differently. Academic datasets accelerate development, whereas commercial efforts emphasize cross-site validation for clinical deployment [7,13]. Dataset shift remains common, with models showing performance declines when tested in new hospitals [13]. The “black box” problem also undermines clinician trust, as binary outputs lack interpretability. Visualization tools such as heatmaps and saliency maps can provide insight, but they are not universally reliable; for example, sometimes highlighting irrelevant regions such as prosthetic hardware or image borders instead of the actual fracture line, potentially misleading clinicians [18]. Integration challenges include picture archiving and communication system (PACS)/radiology information system (RIS) compatibility, workflow delays, and clinician resistance due to medico-legal concerns [19]. At the same time, positive examples exist where systems are fully integrated into PACS platforms and improve throughput. However, evidence for true patient-centered outcomes beyond diagnostic accuracy and workflow efficiency remains scarce [9]. Potential outcomes worth evaluating include cost-effectiveness, reduction in unnecessary follow-up imaging, earlier definitive treatment, and improved functional recovery. Framing validation studies around these endpoints will help determine whether AI ultimately translates into measurable patient benefit.

Pathway to safe integration

To achieve safe clinical integration, use cases should be prioritized where the evidence base is strongest, such as wrist, ankle, and other extremity fractures, before expanding to anatomically more complex sites [5,7]. While some commercial tools (e.g., BoneView) have demonstrated promising performance across a wide array of fracture locations, variation in external settings highlights the value of local validation. External validation is therefore not about questioning regulatory approval, but about confirming consistent performance across different patient populations, scanners, and workflows [17]. AI must function as an assistive tool rather than a replacement [8]. Continuous post-deployment monitoring of sensitivity, specificity, and emergency department outcomes ensures long-term safety [9]. In addition to regulatory approval, alignment with data privacy frameworks and ongoing compliance monitoring are critical to sustain trust and accountability [10,12].

Future directions

The future of AI in fracture detection lies in more advanced approaches. Multi-modal models that integrate radiographs with CT, clinical data, and laboratory results could provide more comprehensive assessments and reduce false interpretations [6]. Adaptive and continual learning frameworks allow AI to recalibrate with evolving datasets, preserving accuracy across different clinical environments [20]. Pediatric-specific models require refinement to account for growth plate variability and bone development. These studies show encouraging diagnostic accuracy, but performance varies across anatomical regions and fracture types, underscoring the need for further refinement. For bone age assessment, AI has also demonstrated improvements in accuracy and consistency across different levels of clinical experience. Future work should focus on validating pediatric-specific models across diverse populations and anatomical sites to ensure robustness and equity.

Equally important is explainability, as transparent AI fosters clinician trust. Techniques such as heatmaps and case-based reasoning are becoming more common, making AI outputs easier to interpret [8]. Further evidence of patient-centered benefits, including reduced treatment delays, fewer complications, and lower healthcare costs, will be best established through retrospective evaluations, pragmatic real-world studies, and post-market surveillance, which avoid the ethical challenges of randomized clinical trials [9]. Finally, widespread adoption will depend on reimbursement pathways, regulatory harmonization, and integration into value-based healthcare systems, as demonstrated by NICE’s draft guidance [12].

## Conclusions

Artificial intelligence in fracture diagnosis has evolved from a promising concept to a maturing clinical tool. The literature consistently shows that AI is most effective when applied as an adjunct in extremity fracture detection, where its diagnostic accuracy approaches that of radiologists and its use improves efficiency in busy emergency settings. However, important limitations remain. Most systems are trained on single-institution datasets, which restricts generalizability and contributes to performance declines when tested externally. Interpretability tools, such as heatmaps, can support clinicians but are not always reliable, and evidence for truly patient-centered outcomes, such as reduced downstream imaging, earlier initiation of treatment, or cost-effectiveness, remains limited.

The main learning points from this review are that AI consistently enhances reader sensitivity and efficiency when used as an assistive tool; regulatory approvals not only confirm clinical promise but also emphasize the need for post-market surveillance; and future priorities include multicenter benchmark datasets, refinement of pediatric-specific models, and systematic evaluation of patient-centered outcomes. Taken together, these insights highlight both the promise and the current gaps in AI for fracture diagnosis. Achieving safe, equitable, and reliable integration will require continued refinement, rigorous validation, and strong collaboration among clinicians, engineers, policymakers, and patients.
